# Objective evaluation of fatigue by EEG spectral analysis in steady-state visual evoked potential-based brain-computer interfaces

**DOI:** 10.1186/1475-925X-13-28

**Published:** 2014-03-12

**Authors:** Teng Cao, Feng Wan, Chi Man Wong, Janir Nuno da Cruz, Yong Hu

**Affiliations:** 1 Department of Electrical and Computer Engineering, University of Macau, Macau, China; 2 Department of Orthopaedics and Traumatology, The University of Hong Kong, Pokfulam, Hong Kong, China

**Keywords:** Fatigue, Objective evaluation, Brain-computer interfaces, Steady-state visual evoked potential, Electroencephalography spectral analysis

## Abstract

**Background:**

The fatigue that users suffer when using steady-state visual evoked potential (SSVEP)-based brain-computer interfaces (BCIs) can cause a number of serious problems such as signal quality degradation and system performance deterioration, users’ discomfort and even risk of photosensitive epileptic seizures, posing heavy restrictions on the applications of SSVEP-based BCIs. Towards alleviating the fatigue, a fundamental step is to measure and evaluate it but most existing works adopt self-reported questionnaire methods which are subjective, offline and memory dependent. This paper proposes an objective and real-time approach based on electroencephalography (EEG) spectral analysis to evaluate the fatigue in SSVEP-based BCIs.

**Methods:**

How the EEG indices (amplitudes in δ, θ, α and β frequency bands), the selected ratio indices (θ/α and (θ + α)/β), and SSVEP properties (amplitude and signal-to-noise ratio (SNR)) changes with the increasing fatigue level are investigated through two elaborate SSVEP-based BCI experiments, one validates mainly the effectiveness and another considers more practical situations. Meanwhile, a self-reported fatigue questionnaire is used to provide a subjective reference. ANOVA is employed to test the significance of the difference between the alert state and the fatigue state for each index.

**Results:**

Consistent results are obtained in two experiments: the significant increases in α and (θ + α)/β, as well as the decrease in θ/α are found associated with the increasing fatigue level, indicating that EEG spectral analysis can provide robust objective evaluation of the fatigue in SSVEP-based BCIs. Moreover, the results show that the amplitude and SNR of the elicited SSVEP are significantly affected by users’ fatigue.

**Conclusions:**

The experiment results demonstrate the feasibility and effectiveness of the proposed method as an objective and real-time evaluation of the fatigue in SSVEP-based BCIs. This method would be helpful in understanding the fatigue problem and optimizing the system design to alleviate the fatigue in SSVEP-based BCIs.

## Background

Brain-computer interfaces (BCIs) provide a direct communication pathway connecting human brain and external devices that is independent to normal peripheral nervous and muscular systems. BCIs measure the brain activities and translate the encoded human intentions to control computers, wheelchairs and robots, with a wide range of applications [1]. Among the non-invasive BCIs, steady-state visual evoked potential (SSVEP)-based BCIs have attracted much attention owing to their merits such as high information transfer rate (ITR), small number of electrodes and short training time in comparison with other types of BCIs [2].

On the other hand, most SSVEP-based BCIs remain in the laboratory demonstration stage, with a big gap from practical use by paralyzed patients or normal users for a long period. One of the challenges is the fatigue that users suffer when using SSVEP-based BCIs. In laboratory demonstrations, the experiment is usually short, e.g., few seconds for one trial or few minutes for one experiment. For a longer period under normal use, when staring at the flashing stimulus however most users have uncomfortable and unpleasant feelings including tiredness, drowsiness, loss of attention and difficulty in concentration, which are the symptoms of fatigue.

Fatigue is generally referred as a feeling of tiredness, reduced alertness, exhaustion, which impairs both capability and willing to perform a task [3-5]. An ideal BCI should be easy to perform with little effort to prevent fatigue but strong electroencephalography (EEG) signals to produce reliable and effective output [6]. Unfortunately, in SSVEP-based BCIs significant mental effort is required for the users to concentrate on the visual stimulus to generate sufficiently strong SSVEP, however due to high brightness, overstimulation and repetitive task, users may easily get fatigue [7]. As attention dependent systems, SSVEP-based BCIs require target gazing and therefore the amplitude and signal to noise ratio (SNR) of the elicited SSVEPs are heavily affected by mental states, fatigue, arousal and degree of attention level [8-10]. Loss of attention and decreased arousal level caused by mental fatigue or distraction can significantly worsen the SSVEP signal quality [11] and consequently degrade the BCI system performance.

In the literature of SSVEP-based BCIs, fatigue and its influence in long-term experiments have been already noticed [12,13], however so far there is no systematic study on the fatigue problems. A frequently used strategy to reduce fatigue is to adopt some relatively comfortable stimulations, such as high frequency stimulation [14,15], high duty cycle stimulation [16] and amplitude modulated stimulation [17]. Unfortunately, the crux of the matter is that there is no objective method to measure and evaluate the fatigue in a reasonable manner. In the existing work, a common choice to “measure” fatigue is self-reported questionnaires provided to the users for feedback about the feelings of fatigue in operating the systems [15,18-20], which are subjective and cannot be done in real time.

When users feel fatigue, they usually have excessive feeling of tiredness and reduced alertness, and also have difficulty in concentrating their attention on the task to perform [4,21]. In the case of decreased attention, arousal level and the reduced capacity to perform the task, the complex synchronization–desynchronization patterns in the 4–13 Hz band can be assumed disrupted [5,22], which are associated with reduced cortical arousal (i.e. EEG shifts from high frequency and low amplitude waves to low frequency and high amplitude ones) [22,23]. More specifically, the decreased attention and arousal level, as well as the reduced capacity are associated with the global increases in the θ and α activities [22,23]. The θ activity occurs in a variety of mental states including drowsiness, and the increase in θ activity is related to generalized performance decrements on task [22]. The α waves appear during relaxed conditions, at decreased attention levels and in a drowsy but wakeful state [22,24], and the increased α power associated with fatigue is related to the increased mental effort to maintain vigilance level [22]. Since the EEG reflects the mental state and fatigue of the users, EEG spectral analysis could be a promising method to provide an objective and real-time evaluation of the fatigue level in SSVEP-based BCIs.

Our preliminary study [25] shows that the increased θ, α, and (θ + α)/β power are associated with the increased fatigue level, and the SSVEP amplitude and SNR are influenced by fatigue level. On this basis, this paper further checks the feasibility and effectiveness of proposed objective method for fatigue measure and evaluation in SSVEP-based BCIs through two elaborate SSVEP-based BCI experiments, one validates mainly the effectiveness and one considers more practical situations. The changes of EEG indices (δ, θ, α and β), ratio indices (θ/α and (θ + α)/β), and SSVEP properties (amplitude and SNR) associated with increasing fatigue level are investigated. The proposed method would be helpful in understanding the fatigue problem from the viewpoint of EEG spectrum. With appropriate methods for measure and evaluation, a systematic study on the influence due to different configurations (such as different stimulus frequencies, colors and patterns) can be performed, and eventually the design of the system including the visual stimulator can be optimized in order to alleviate the fatigue in SSVEP-based BCIs.

## Methods

### Experiment design

In order to prove the feasibility and effectiveness of the proposed method, two elaborate SSVEP-based BCI experiments were designed and two different groups of participants were tested in this study. More specifically, in Experiment 1 a single stimulus was used to elicit SSVEP at the selected frequency and the purpose was to prove that the EEG spectral analysis is effective in measuring the fatigue in SSVEP-based BCIs; Experiment 2 employed several stimuli encoded by different frequencies for the purpose to show the feasibility of the proposed method in practical situation, because in a real SSVEP-based BCI, normally there must be a number of buttons for different commands presented by different stimuli with different frequencies, rather than only one stimulus as in Experiment 1.

Twenty-one randomly selected university students (aged from 21 to 29 years old) participated in this study. The participants were selected according to the following criteria: no history of psychiatric or neurological disorders, no psychotropic medications or addiction drugs and with normal or corrected to normal vision. Informed written consent was obtained from all participants after explaining the nature, possible consequences and privacy issues of the study to them. The protocol was in accordance with the Declaration of Helsinki and approved by the Research Ethics Committee (University of Macau).

An LCD monitor was used as the visual stimulator (ViewSonic 22”, refresh rate 120 Hz, 1680×1050 pixel resolution), which was programmed with Microsoft Visual Studio 2010 and Microsoft DirectX SDK (June 2010). White stimuli with 120×120 pixels on black background were used for best contrast in this study. There was a symbol “+” shown in the center of the stimulus before and within each trial, indicating the stimulus that the participants should gaze at and keeping the participants focusing on the target, so the participants could follow the cues to gaze at different stimuli. The experiment facilities were set in a normal office environment without intensive light and noise, and participants were seated on a comfortable chair in front of the visual stimulator with a distance of about 50 cm. Since the participants were told to keep the body unmoved and not to blink eyes in each trial, the artifacts and other noises were controlled to the minimum. All the experiments were conducted in a fixed time period (3–4 p.m.), and all the participants finished the experiments successfully without suffering any unpleasant feeling and discomfort in bodies.

### EEG measurement

A standard EEG electrode placed on O_Z_, in the international 10–20 montage system, which was located in the center of the occipital lobe, was used as the input channel. The reference electrode and ground electrode were chosen as FCz and Cz, respectively. EEG signals were amplified through an amplifier (g.USBamp, Guger Technologies, Graz, Austria) and filtered by a 50 Hz notch filter and a 0.5 Hz to 60 Hz band-pass filter to remove the noise. The sampling frequency was 600 Hz. The frequency bands δ (1–4 Hz), θ (4–8 Hz), α (8–13 Hz), and β (13–30 Hz) were monitored along the experiments.

### Psychological measurement

All the participants were asked to finish a self-reported fatigue questionnaire before and after the task, which was based on the Chalder Fatigue Scale (CFS) [26]. The CFS had high reliability and validity and consisted of 8 physical fatigue questions and 6 mental fatigue questions, such as “Do you have problems with tiredness?”, “Do you need to rest more?” and “Do you feel sleepy or drowsy?” Besides, another 6 questions related to visual fatigue symptoms were added in this questionnaire. These six questions were: “Do you have eyestrain?” “Do you have eye dryness?” “Do you have headache?” “Do you feel fatigue in eyes?” “Do you feel blurry?” and “Do you have difficulties keeping eyes open?” Therefore, there were 20 questions in total and every question had four choices rated as a four-point scale (0–3), i.e. better than usual (0), no more than usual (1), worse than usual (2) and much worse than usual (3). A high fatigue score presented a high level of fatigue, and in this study the questionnaire was used to provide a subjective reference to evaluate users’ fatigue level before and after experiments.

### Experiment procedure

In the first SSVEP-based BCI experiment, only one stimulus flashing at 15 Hz was used to elicit SSVEP. Eight participants participated in this experiment and were asked to gaze at the flashing stimulus for 30 trials. The stimulus was flashing for 3 seconds in each trial with a 2-second pause for a short rest between two consecutive trials.

The paradigm of the second experiment employed several stimuli encoded by different frequencies and thus simulated a more general and practical situation. There were six stimuli distributed in two rows and three columns in this experiment. Thirteen participants participated in this experiment and they were asked to gaze from the stimulus 1 to the stimulus 6, and repeated for 6 times (i.e. 36 trials in total). To test on the commonly used stimulus frequencies, two sessions were performed, with the stimulation frequencies in 7–12 Hz for Session I and 13–18 Hz for Session II. The stimuli were flashing for 8 seconds in each trial with a 4-second pause between two consecutive trials. Every participant did the experiment for both two sessions and a 5-minute pause was given between the sessions to reduce the fatigue caused by the previous one.

### Data analysis

Since the stimulation frequencies for each trial were already known, it was easy to calculate the amplitudes of δ, θ,α and β frequency bands, as well as the SSVEP amplitude and SNR at the stimulation frequency by Fast Fourier Transform (FFT) using all the sampling points in each trial. The SNR was defined as the ratio of the amplitude at stimulation frequency to the mean value of the n adjacent points:

(1) $$\mathrm{SNR}=\frac{n\times y\left(f\right)}{\sum_{k=1}^{n/2}\left[y\left(f+0.25\times k\right)+y\left(f-0.25\times k\right)\right]}$$

where *y* was the amplitude spectrum calculated by FFT, *f* was the stimulation frequency, and *y*(*f*) was the SSVEP amplitude at stimulation frequency (refer to [27] for more details). When calculating the amplitudes of δ, θ, α and β frequency bands, the elicited SSVEP component at the stimulation frequency had been filtered from the corresponding frequency band by a band-stop filter. The mean value of the amplitudes in the frequency band was selected as the index of this frequency band and used to calculate the ratio indices, such as θ/α and (θ + α)/β that were two widely used ratio indices in fatigue studies [24,28-30]. In total, four EEG indices (δ, θ, α and β), two ratio indices (θ/α and (θ + α)/β), and two SSVEP indices (amplitude and SNR) in each trial along the experiments were obtained. The mean values of each index in the first five trials and the last five trials in the experiment were used to represent the corresponding index in the alert state and the fatigue state, respectively. The fatigue scores assessed by the fatigue questionnaire before and after the experiment were considered as the reference fatigue scores in the alert and fatigue states. One-way ANOVA with significance level of 0.05 was employed to test the significance of the difference between the alert state and the fatigue state for each index.

## Results

### Experiment 1: single stimulus

**Figure 1:**
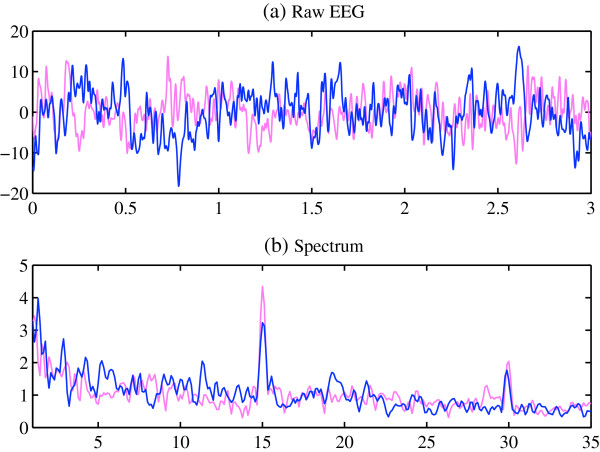
**Comparison of Raw EEG signal and the spectrum of an individual participant between the alert and fatigue states for Experiment 1.** Vertical axis for **(a)** and **(b)**, amplitude (μV); horizontal axis for **(a)**, time (sec); horizontal axis for **(b)**, frequency (Hz); red curve: alert state; blue curve: fatigue state.

**Figure 2:**
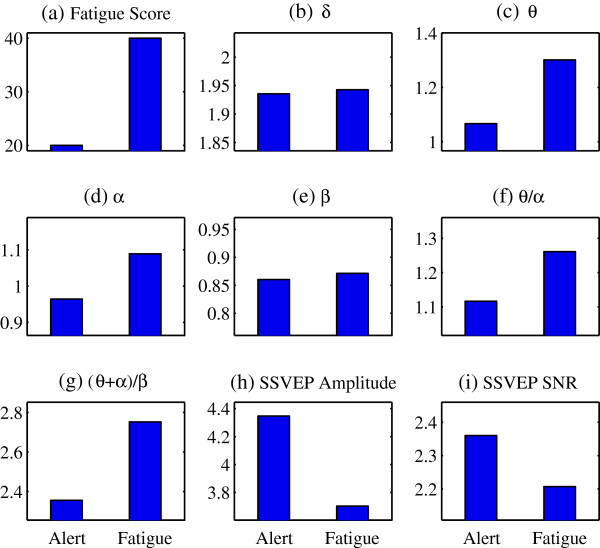
**Comparison of indices of an individual participant between the alert and fatigue states for Experiment 1.** Comparison of the average values and SD of fatigue score, EEG indices, ratio indices, as well as SSVEP amplitude and SNR in the alert and fatigue states. Vertical axis for **(b)**-**(i)**, amplitude (μV); horizontal axis for **(a)**-**(i)**, Alert: alert state, Fatigue: fatigue state.

First, the result of each individual was analyzed separately. Figure 1 shows the comparison of the raw EEG signals and the spectrums of a participant between the alert and fatigue states in Experiment 1. Since the stimulation frequency was 15 Hz, the SSVEP components can be found at the fundamental frequency 15 Hz and the harmonic frequency 30 Hz in both alert and fatigue states. It is worth notice that the amplitude of SSVEP component at the fundamental frequency 15 Hz was decreased along the time elapsed. In Figure 1(b), it is difficult to distinguish two curves for the alert and fatigue states from the spectrums, despite some slight increases of the amplitude in 4–14 Hz frequency range that covered the θ and α bands. In order to discover the difference between the alert and fatigue states, different EEG indices were calculated. Figure 2 presents the comparison of the fatigue scores from self-reported questionnaire and different indices in the alert state and the fatigue state for this participant. The fatigue score by self-reported questionnaire was increased from 20 to 40, which suggests the increased fatigue level due to the experiment. The changes are obvious in the indices θ and α, as well as the ratio indices θ/α and (θ + α)/β resulting from the fatigue. In addition, Figure 2(h) and Figure 2(i) indicate the reduced amplitude and SNR of the elicited SSVEP in the fatigue state compared with the alert state.

**Figure 3:**
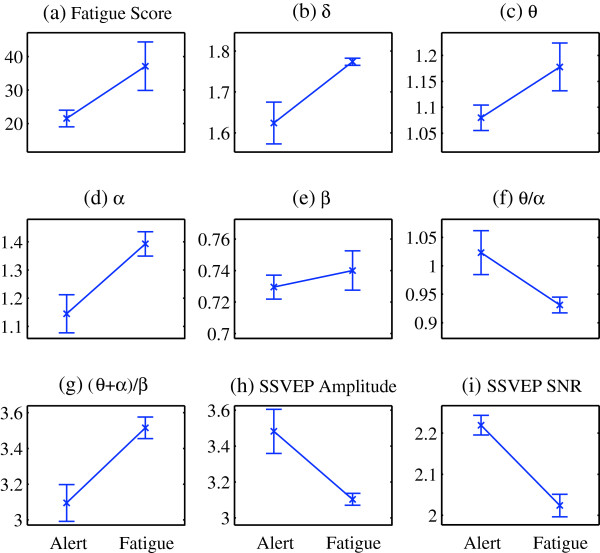
**Comparison of indices between the alert and fatigue states for Experiment 1.** Comparison of the average values and SD of fatigue score, EEG indices, ratio indices, as well as SSVEP amplitude and SNR in the alert and fatigue states. Vertical axis for **(b)**-**(i)**, amplitude (μV); horizontal axis for **(a)**-**(i)**, Alert: alert state, Fatigue: fatigue state.

Besides the individual case study, an overall analysis on all participants was also performed. The self-reported fatigue questionnaire shows that in Experiment 1 the participants’ average fatigue level was significantly increased after the experiment. The fatigue score was significantly increased (*F* (1, 14) = 33.11, p < 0.001), with pre-mean score = 21.50, standard deviation (SD) = 2.51; and post-mean score = 37.13, SD = 7.36. Figure 3 demonstrates the comparison of the fatigue scores and indices in the alert state and the fatigue state. Four EEG indices δ, θ, α, β and the ratio index (θ + α)/β show an increasing tendency, while the ratio index θ/α, SSVEP amplitude and SNR show an opposite tendency. The statistical test results of the indices are given in Table 1. Significant increases are found in δ (*F* (1, 8) = 41.77, *p* < 0.001), θ (*F* (1, 8) = 17.72 *p* < 0.001), α (*F* (1, 8) = 47.64, *p* < 0.001), and the ratio index (θ + α)/β (*F* (1, 8) = 66.40, *p* < 0.001), while significant decreases are observed in the ratio index θ/α (*F* (1, 8) = 22.89, *p* < 0.001), as well as the SSVEP amplitude (*F* (1, 8) = 41.32, *p* < 0.001) and SNR (*F* (1, 8) = 145.29, *p* < 0.001). The β index (*F* (1, 8) = 2.59, *p* = 0.146) has no significant difference between the alert state and the fatigue state. Nevertheless, due to the individual differences, different sensitivities were found on the increasing fatigue level, so the changes of different indices could be different among participants. That is why the θ/α index was found increased in the individual case presented in Figure 2(f), while the group average was decreased as shown in Figure 3(f).

**Table 1:** Statistical test results for Experiment 1

**Index**	**Alert Mean (SD)**	**Fatigue Mean (SD)**	**ANOVA**		**Change direction**
			***F***	***p***	
δ	1.62 (0.05)	1.77 (0.01)	41.77	<0.001	↑
θ	1.08 (0.02)	1.18 (0.04)	17.72	<0.001	↑
α	1.15 (0.07)	1.39 (0.04)	47.64	<0.001	↑
β	0.73 (0.01)	0.74 (0.01)	2.59	=0.146	―
θ/α	0.94 (0.05)	0.84 (0.01)	22.89	<0.001	↓
(θ + α)/β	3.05 (0.10)	3.48 (0.07)	66.40	<0.001	↑
SSVEP amplitude	3.48 (0.12)	3.11 (0.03)	44.32	<0.001	↓
SSVEP SNR	2.22 (0.02)	2.02 (0.03)	145.29	<0.001	↓

Alert Mean: average value of the first five trials in the experiment; Fatigue Mean: average value of the last five trials in the experiment. SD: standard deviation; ↑: increase; ↓: decrease; ―: unchanged; degrees of freedom for the ANOVA = (1,8).

### Experiment 2: multi-stimulus with different frequencies

**Figure 4:**
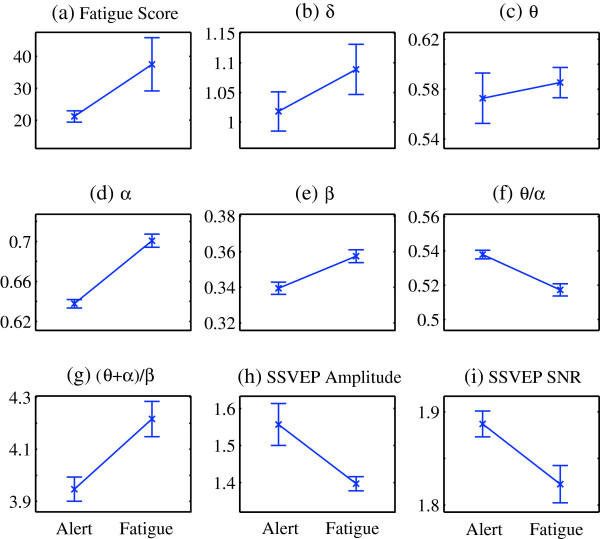
**Comparison of indices between the alert and fatigue states for Experiment 2.** Comparison of the average values and SD of fatigue score, EEG indices, ratio indices, as well as SSVEP amplitude and SNR in the alert and fatigue states. Vertical axis for **(b)**-**(i)**, amplitude (μV); horizontal axis for **(a)**-**(i)**, Alert: alert state, Fatigue: fatigue state.

In Experiment 2, as assessed by the self-reported fatigue questionnaire, the participants’ average fatigue level was found significantly increased after the experiment: the fatigue score was significantly increased (*F* (1, 14) = 124.72, *p* < 0.001), with pre-mean score = 21.17, SD = 1.75, and post-mean score = 37.50, SD = 8.34. Figure 4 demonstrates the comparison of the fatigue scores and indices in the alert state and the fatigue state. The change trends are quite similar as the results obtained in Experiment 1: four EEG indices δ, θ, α, β and the ratio index (θ + α)/β exhibit an increasing tendency, while the ratio index θ/α, SSVEP amplitude and SNR show an opposite tendency. The statistical test results of the indices are given in Table 2. Significant increases are found in δ (*F* (1, 8) = 8.70, *p* <0.05),α (*F* (1, 8) = 326.07, *p* < 0.001), β (*F* (1, 8) = 64.25, *p* < 0.001) and the ratio index (θ + α)/β (*F* (1, 8) = 53.38, *p* < 0.001), while significant decreases are found in the ratio index θ/α (*F* (1, 8) = 83.34, *p* < 0.001), as well as the SSVEP amplitude (*F* (1, 8) = 35.88, *p* < 0.001) and SNR (*F* (1, 8) = 35.40, *p* < 0.001). There is no significant difference in θ (*F* (1, 8) = 1.42, *p* = 0.267) between the alert state and the fatigue state.

**Table 2:** Statistical test results for Experiment 2

**Index**	**Alert Mean (SD)**	**Fatigue Mean (SD)**	**ANOVA**		**Change direction**
			***F***	***p***	
δ	1.02 (0.03)	1.09 (0.04)	8.70	<0.05	↑
θ	0.57 (0.02)	0.59 (0.01)	1.42	=0.267	―
α	0.64 (0.01)	0.70 (0.01)	326.07	<0.001	↑
β	0.34 (0.01)	0.36 (0.01)	64.25	<0.001	↑
θ/α	1.04 (0.02)	0.97 (0.01)	84.34	<0.001	↓
(θ + α)/β	3.95 (0.05)	4.21 (0.07)	53.38	<0.001	↑
SSVEP amplitude	1.56 (0.06)	1.39 (0.02)	35.88	<0.001	↓
SSVEP SNR	1.97 (0.03)	1.84 (0.04)	35.40	<0.001	↓

Alert Mean: average value of the first five trials in the experiment; Fatigue Mean: average value of the last five trials in the experiment. SD: standard deviation; ↑: increase; ↓: decrease; ―: unchanged; degrees of freedom for the ANOVA = (1,8).

## Discussion

### Summary of evaluation results

#### *Experiment 1: single stimulus*

The users’ fatigue level is found significantly increased after the experiment assessed by the fatigue questionnaire. The statistical test results demonstrate that the indices δ, θ and α have significant differences between the alert state and the fatigue state. These results suggest that the increases in δ, θ and α are associated with the increasing fatigue level along the SSVEP experiments. For the ratio indices θ/α and (θ + α)/β, both of them show significant differences between the alert state and the fatigue state. Therefore, the decrease in θ/α and the increase in (θ + α)/β are associated with the increasing fatigue level. Moreover, the experiment results indicate that both the amplitude and the SNR of the elicited SSVEPs are significantly decreased by the increasing fatigue level.

#### *Experiment 2: multi-stimulus with different frequencies*

The users’ fatigue level is significantly increased after the experiment assessed by the fatigue questionnaire. The statistical test results demonstrate that the indices δ, α and β have significant differences between the alert state and the fatigue state. The increases in δ, α and β are associated with the increasing fatigue level along the SSVEP experiments. Regarding the change of ratio indices θ/α and (θ + α)/β, both of them show significant differences between the alert state and the fatigue state. Consistent with the results in Experiment 1, the decrease in θ/α and increase in (θ + α)/β are associated with the increasing fatigue level. Moreover, the experiment results show that the amplitude and the SNR of the elicited SSVEPs are significantly decreased by the increasing fatigue level, which are also consistent with the results in Experiment 1.

### Feasibility and effectiveness of evaluation on fatigue by EEG spectral analysis

It is found that most of the results are consistent in two elaborate SSVEP-based BCI experiments with two different groups of participants. The significant increases in δ and α are found associated with the increasing fatigue level in both experiments. For the θ and β indices, even the results are not consistent in two experiments, it is still observed that they have the same trends. For the ratio index θ/α, because the change in α is more significant than the change in θ, the significant increase in θ/α is obtained and is associated with the increasing fatigue level in both experiments. For the ratio index (θ + α)/β, the significant increase is found associated with the increasing fatigue level in both experiments. Since θ and α have the same tendency while the change in β is smaller compared with the changes in θ and α, (θ + α)/β amplifies the difference between θ and α, which is sensitive in evaluating the fatigue. Despite some variations due to different settings in the two paradigms and individual differences, significant results are found in both experiments with the α, θ/α and (θ + α)/β indices. This demonstrates that EEG spectral analysis can provide consistent objective evaluation of the fatigue in SSVEP-based BCIs.

Moreover, it is found that the amplitude and the SNR of the elicited SSVEPs are significantly affected by the increasing fatigue level. The results support that the amplitude and SNR of the elicited SSVEPs are easily affected by mental states, fatigue, and degree of attention level. As significant mental effort is required to elicit SSVEP with high amplitude and SNR, the participants get fatigue easily. The decrement of arousal and the loss of attention caused by significant mental effort and repetitive stimulation will reduce the quality of the elicited SSVEPs. It is more difficult to elicit good quality SSVEPs in the fatigue state even the participants are still focusing on the targets. Furthermore, the decreases of the amplitude and the SNR of the elicited SSVEPs consequently deteriorate the discrimination accuracy, detection speed, ITR and overall performance of the BCI systems.

### Physiological explanation

Some studies showed that the δ wave is significantly increased when participants are getting fatigue [31,32]. The δ wave is the lowest brain activity which is usually associated with deep stages of sleep, and it is also a pathological slow wave and associated with a wide array of disorders [33]. When a participant is getting fatigue, the drowsiness and decrease arousal level will elevate slow wave brain activities. However, the δ wave is easily influenced by artifacts which usually invade low EEG frequencies. In addition, all the participants in the experiments are in awake state and do not have any type of disorders. Therefore, it is not sure the significant increase in δ is associated with increasing fatigue level.

The increased θ wave was found associated with increasing fatigue level in many previous fatigue monitoring studies [5,31,32,34,35]. The θ wave is related to sleep, work memory, cognitive performance and a variety of mental states [22,33]. Increased θ activity has been proven associated with generalized performance decrements, including working memory, information encoding and so forth [22]. When a user is getting fatigue along the experiment, the user will feel drowsy and tired with the capacity degraded due to the loss of attention and decrease of arousal.

The results in α wave were consistent with the existing studies showing that the significant increase in α activity is associated with the increasing fatigue level [5,24,31,34,35]. The α wave appears in the relaxed and effortless alertness mental state, and gives a relevant indication about the occurrence of a state of relaxation or of low arousal [33,34]. The increased α activity is associated with an increased mental effort to maintain vigilance level [22]. When participants are getting fatigue, their attention, concentration and vigilance level will decrease. Therefore, the participants need to put more attention and increase mental effort to focus on the target and maintain the vigilance level in the fatigue state compared with the alert state.

The increased β wave is associated with increasing alertness, arousal and excitement [33]. In this study, the β activity shows an increasing trend over time. Previous studies about the association between β wave and fatigue are quite variable: the β activity had been found altered in [36] with the increasing fatigue level, other studies reported significant increases in β wave [5] and decreases in β [24] associated with increasing fatigue level, but no significant change in β according to other two literatures [32,37].

Similar results were also obtained in other fatigue studies with the ratio index (θ + α)/β. In [24], (θ + α)/β index showed a significant increase associated with the increasing fatigue level, and also related to the mental alertness level. However, no significant change was observed in θ/α in this research. A big increase was found in (θ + α)/β at the end of task in [28]. The θ and α activities are already proved to be associated with the fatigue, while the relation between β wave and the fatigue are quite variable. In this study, the change in θ and α are more significant than the change in β, the ratio index (θ + α)/β thus amplifies the mutual addition effects from θ and α. Moreover, increased θ activity is found associated with the decreased generalized performance and arousal level, and the increased α band is associated with an increased mental effort to maintain vigilance level. Therefore, the decrease of θ/α indicates that the user is putting more attention and mental effect to focus on the target and fight against the increasing fatigue level. In this study, two widely used ratio indices θ/α and (θ + α)/β are adopted to measure the fatigue, as this is helpful to the comparison of the findings with the results in the literature of fatigue research. Other choices on the indices are possible and deserve further study.

## Conclusions

In this paper, an objective approach based on EEG spectral analysis is proposed to evaluate the fatigue in SSVEP-based BCIs. In order to prove the feasibility and effectiveness of the proposed method, two elaborate SSVEP-based BCI experiments are designed and tested with two different groups of participants. Consistent experiment results indicate that the significant increases in α and (θ + α)/β, as well as the decrease in θ/α are associated with the increasing fatigue level. In addition, the experiment results also show that the amplitude and SNR of the elicited SSVEPs are significantly affected by users’ fatigue in the SSVEP-based BCI experiments. The proposed approach would be promising in providing an objective, quantitative and real-time measure of the fatigue in SSVEP-based BCIs. With such a measure a systematic study on the factors such as stimulus frequency, color and duty cycle, as well as their influences on fatigue in SSVEP-based BCIs could be performed, which would be helpful in understanding the fatigue problem and eventually the design of optimal SSVEP-based BCIs with the fatigue alleviated.

## Competing interests

The authors declare that they have no competing interests.

## Authors’ contributions

FW and TC initiated the idea and conceived the study. TC, CMW and JNC designed and conducted experiments. TC performed data analysis, and drafted the manuscript. FW and YH supervised, revised and gave the final approval of the manuscript. All authors read and approved the final manuscript.
